# Impact of modifiable healthy lifestyle adoption on lifetime gain from middle to older age

**DOI:** 10.1093/ageing/afac080

**Published:** 2022-05-11

**Authors:** Ryoto Sakaniwa, Midori Noguchi, Hironori Imano, Kokoro Shirai, Akiko Tamakoshi, Hiroyasu Iso, The JACC Study Group

**Affiliations:** Public Health, Department of Social Medicine, Osaka University Graduate School of Medicine, Suita, Osaka 565-0871, Japan; Public Health, Department of Social Medicine, Osaka University Graduate School of Medicine, Suita, Osaka 565-0871, Japan; Public Health, Department of Social Medicine, Osaka University Graduate School of Medicine, Suita, Osaka 565-0871, Japan; Department of Public Health, Kindai University Faculty of Medicine, Osakasayama 589-8511, Japan; Public Health, Department of Social Medicine, Osaka University Graduate School of Medicine, Suita, Osaka 565-0871, Japan; Public Health, Department of Social Medicine, Hokkaido University Graduate School of Medicine, Sapporo, Hokkaido 060-8638, Japan; Public Health, Department of Social Medicine, Osaka University Graduate School of Medicine, Suita, Osaka 565-0871, Japan

**Keywords:** healthy lifestyle, lifestyle factors, life expectancy, older people

## Abstract

**Objective:**

this study explored whether the modification of selected lifestyles is likely to increase life expectancy from middle age onwards, regardless of the presence of major comorbidities.

**Methods:**

we examined a prospective cohort of 20,373 men and 26,247 women aged 40–80 years. Eight modifiable lifestyle factors were assessed: consumption of fruit, fish and milk, walking and/or sports participation, body-mass index, smoking status, alcohol consumption and sleep duration. Modifiable healthy lifestyle factors scored one point each, for a maximum of eight points. The impact of modifiable healthy lifestyle adoption on lifetime gain during the ages of 40–102 years was analysed.

**Findings:**

during the median 21 years of follow-up, 8,966 individuals (3,683 men and 5,283 women) died. Life expectancy at 40 years (95% confidence intervals) for 7–8 health lifestyle points was 46.8 (45.6–48.1) and 51.3 (50.0–52.6) years for men and women, respectively. The potential impact of modifiable healthy lifestyle adoption on lifetime gain persisted over the age of 80 years or more, in individuals with ≥5 factors (*P* < 0.001), particularly older men. The benefits were more pronounced among patients with major comorbidities, such as cardiovascular disease, cancer, hypertension, diabetes, kidney disease and those with multimorbidity throughout all age categories.

**Conclusion:**

adopting modifiable healthy lifestyles was associated with lifetime gain, even in individuals aged 80 years or more, regardless of the presence of any major comorbidities in each life stage since middle age. The findings imply the importance of improving the one’s lifestyle for an increased lifespan, even among older patients and/or those with multimorbidity.

## Key Points

- Benefits of healthy lifestyle adoption for lifetime gain persist in people aged 80 years or more, particularly men.
- The benefits were more pronounced in patients with major comorbidities, such as cancer and cardiovascular disease, and those with multimorbidity throughout all age categories.
- Improving the one’s lifestyle for an increased lifespan is important even among older patients and/or those with comorbidity.

## Introduction

Life expectancy has improved globally [1], and the average life expectancy in many industrialised countries is expected to be 85 years or more by 2030 [2, 3]. A prolonged lifetime is attributed to social factors, such as sociodemographic status [1], gross domestic product per capita [4, 5], healthcare expenditure and universal health coverage [6, 7] and policies and legislation [8]. Meanwhile, the joint impact of selected modifiable healthy lifestyle factors, such as moderate physical activity [9], healthy body-mass index (BMI) [10], non-smoking status [11, 12], moderate alcohol intake [13, 14] and appropriate sleep duration [15, 16] has been associated with an increased lifespan in industrialised countries [17–23]. These studies suggest that adopting a modifiable healthy lifestyle may individually improve longevity.

However, multiple controversial arguments persist. First, as national life expectancy has plateaued in countries with high average life expectancy in recent years [3], it is uncertain whether the benefits of modifiable healthy lifestyle factors are absent in older populations and/or those with higher average life expectancies. Second, evidence is limited for patients with comorbidities and those with multimorbidity. The prevalence of cardiovascular disease (CVD), cancer, hypertension, diabetes and kidney diseases has increased globally and is a major cause of death in adult and older people [24].

This study thus aimed to investigate whether a modifiable healthy lifestyle had increased the lifespan of individuals from middle to old age, regardless of the presence of any major comorbidities at each life stage. We examined the impact of a modifiable healthy lifestyle on lifetime gain at several age categories among over 40,000 Japanese individuals who are considered to have the longest life expectancy worldwide.

## Methods

### Study population

This was a baseline survey of the Japan Collaborative Cohort (JACC) Study, a large prospective study conducted between 1988 and 1990. A total of 110,585 (46,395 men and 64,190 women) participants aged 40–79 years from 45 communities across Japan completed self-administered questionnaires about their lifestyles and medical histories. The sampling methods and protocols of the JACC Study have previously been described in detail [25]. We excluded 27,476 (12,061 men and 15,415 women) participants from 13 of the original 45 communities for whom data on one or more modifiable lifestyle factors were not obtained. We also excluded 34,085 individuals (12,878 men and 21,207 women) who did not have complete information on the eight modifiable healthy lifestyle components discussed below. The data of the remaining 49,021 participants were analysed (21,453 men and 27,568 women). Informed consent was obtained from participants or community leaders. The ethics committees of the Nagoya University School of Medicine and Osaka University approved the protocol of this investigation, as per the Declaration of Helsinki.

### Components of modifiable healthy lifestyles

A self-administered questionnaire measured the eight components of modifiable healthy lifestyles at baseline: consumption of fruits, fish and milk, walking and/or sports participation, BMI, smoking status, sleep duration and alcohol consumption. Points allocated for healthy behaviours were totalled to obtain the modifiable healthy lifestyle scale, ranging from 0 to 8. Justification for the eight selected components, as well as their validity, is described elsewhere [25, 26].

The frequency of consumption of fruit, fish and milk during the preceding year could be indicated as ‘rarely’, ‘1–2 days a month’, ‘1–2 days a week’, ‘3–4 days a week’, and ‘almost every day’. One point each was allocated for fruit ≥ 1/day (≥7/week), fish ≥ 1/day (≥7/week) and milk almost every day. For average daily walking, possible responses were ‘rarely’, ‘0.5 h’, ‘0.5–1 h’ and ‘1 h or more’. Possible responses for average weekly sports participation were ‘rarely’, ‘1–2 h’, ‘3–4 h’ and ‘5 h or more’. One point was allocated for those who selected ‘0.5–1 h’ and ‘1 h or more’ for walking per day, as well as for those participating in sports for ‘5 h or more’ per week. As for BMI, self-reported weight (kg) was divided by the square of self-reported height (m^2^); one point was allocated if an individual’s BMI was 21.0–25.0 kg/m^2^. One point was allocated for current non-smoking, current non-drinking or consuming 1–46.0 g ethanol/day and a sleep duration of 5.5–7.4 h/day.

### Information for all-cause mortality

Mortality data were centralised at the Ministry of Health and Welfare. Furthermore, the underlying causes of death were coded according to the International Statistical Classification of Diseases and Related Health Problems, 10th revised edition (ICD-10). Participants who died after removal from their original communities were treated as censored cases. The end of the final follow-up was 31 December 2009. For the subtypes, I00–I99 was coded as CVD; C00–C98 was cancer or other cause of death. Investigators reviewed death certificates, which were forwarded to the public health centre in the deceased’s area of residency.

### Statistical analysis

Population characteristics according to modifiable healthy lifestyle scores, from 0 to 7–8, were presented as numbers with percentages and means with standard deviations, depending on the nature of the variables. The differences were tested using a one-way analysis of variance (ANOVA) and the chi-squared test where appropriate.

To evaluate the risk of all-cause mortality associated with modifiable healthy lifestyles, we first calculated the impact of each modifiable healthy lifestyle component on all-cause mortality using hazard ratios (HRs) and 95% confidence intervals (95% CI). Next, we examined HRs (95% CI) for all-cause mortality according to the number of modifiable healthy lifestyle components, ranging from 0–2 to 7–8 points.

To calculate sex-specific life expectancy, we used the life-table method, stratified by the number of modifiable healthy lifestyle components. The life tables were created starting at age 40 and ending at age 102 years, with single-year intervals. The prediction for specific lifetime survival probability at age *X* years was determined by sex-specific Gompertz proportional hazard regression for all-cause mortality according to modifiable healthy lifestyle components. To estimate sex-specific remaining life expectancy at age *X* years, we fitted lifetime survival probabilities into life tables, based on a hypothetical cohort of 100,000 at age 40 years, stratified by modifiable healthy lifestyle components.

Survival probability and expected number of deaths were set at 100% at age 40 years. The probability of survival between ages [*X, X* + 1] was calculated based on mortality rate and person-years of survival within [*X, X* + 1]. The 95% CI was estimated by Monte Carlo simulation with 10,000 bootstrapping of samples. The impact of a modifiable healthy lifestyle on lifetime gain during the ages 40–102 years was estimated based on a Bayesian model of Gompertz proportional hazard regression adjusted by educational level and family history of CVD. We also confirmed the impact of cumulated healthy lifestyles on lifetime gains at ages 50, 65 and 80 years among participants with major comorbidity (CVD, cancer, hypertension, diabetes and kidney diseases) and multimorbidity.

To investigate the applicability of our cohort data to a real-world setting, we determined the lifetime survival probability of Japanese men and women using Japanese national census data from 2018 [27]. We examined potential bias by comparing the age-adjusted baseline characteristics, primary results and average life expectancies at ages 50–102 years between the included (20,373 men and 26,247 women) and excluded (*n* = 24,942 for men and 36,622 for women) populations. Furthermore, we examined changes in healthy behaviours between the baseline and 5 years of follow-up. Two-tailed *P* < 0.05 was defined as a significant difference. All statistical procedures were performed using SAS version 9.4 (SAS Institute Inc., Cary, NC). Monte Carlo simulations were performed with @RISK 7.5 (Palisade Corporation, Newfield, NY).

### Role of the funding source

The study funders had no role in the study design, data collection, data analysis, data interpretation or writing of the report.

## Results

Over a median follow-up period of 19.6 years, 49,021 participants (male: 43.7%) were included in the study, with a mean age of 56.8 years at baseline. All-cause mortality reached 9,865 individuals (5,824 men and 4,041 women). The mean remaining life expectancy at age 40 years was 42.7, 49.1 and 46.1 years for men, women and men and women combined, respectively.

Sex-specific baseline characteristics are presented in Table 1. Men with a higher number of modifiable healthy lifestyles were older, whereas rich women were younger. Family history of CVD, educational level and the prevalence of comorbidities were positively associated with the number of healthy lifestyles for both men and women (*P* < 0.002).

**Table 1:** Sex-specific mean values and presentation of baseline risk characteristics according to modifiable healthy lifestyle scores

	Men							Women						
	Number of modifiable heathy lifestyles, points							Number of modifiable heathy lifestyles, points						
**Subjects**	0–2	3	4	5	6	7–8	*P* for trend	0–2	3	4	5	6	7–8	*P* for trend
No. at risk, no.	5,274	5,416	5,317	3,525	1,487	434	–	810	3,010	6,246	7,993	6,297	3,212	–
Age, years (standard deviation)	55.0 (10.2)	55.4 (10.0)	56.0 (9.9)	56.3 (9.9)	56.4 (9.6)	57.3 (9.6)	<0.001	57.8 (11.1)	57.0 (10.7)	56.2 (10.3)	55.8 (9.9)	55.4 (9.4)	55.5 (8.9)	0.002
Family history of cardiovascular disease, %	40.7	39.0	41.8	42.9	44.7	40.3	0.002	40.7	41.2	40.5	40.9	42.6	43.9	0.002
College or higher education, %	16.2	18.7	19.6	20.4	22.4	27.3	<0.001	7.0	8.3	9.6	10.9	13.1	13.4	<0.001
High perceived mental stress, %	25.8	25.1	24.3	25.5	24.5	27.2	0.391	20.4	21.7	20.6	21.0	21.1	21.7	0.545
Fruits >1/day, %	11.8	31.4	51.2	70.7	84.1	94.1	<0.001	5.0	21.2	42.6	65.7	83.2	96.1	<0.001
Fish >1/day, %	9.0	18.1	26.9	40.4	55.0	77.8	<0.001	2.2	4.6	11.3	22.0	38.0	69.8	<0.001
Milk almost every day, %	13.3	30.4	48.3	65.7	81.1	92.0	<0.001	3.7	13.1	28.8	47.4	68.0	88.6	<0.001
Habitual exercise and/or walking, %	28.0	46.3	57.5	67.2	77.3	90.5	<0.001	10.2	23.6	39.1	51.0	67.0	85.0	<0.001
Body-mass index 21–25 kg/m^2^, %	29.1	49.0	60.9	70.6	81.1	91.0	<0.001	7.3	20.5	34.9	51.7	66.9	86.9	<0.001
Ethanol intake <46.0 g/day, %	41.6	65.9	76.8	85.0	92.2	96.7	<0.001	91.4	98.0	99.3	99.6	99.8	99.9	<0.001
Never having smoked, %	5.0	12.8	22.2	35.1	52.5	75.4	<0.001	60.8	84.5	93.0	96.3	98.4	99.6	<0.001
Sleep 5.5–7.4 h/day, %	26.0	46.1	56.2	65.3	76.8	91.0	<0.001	11.2	34.5	51.0	66.3	78.7	91.2	<0.001
**Comorbiditie**s														
Cardiovascular disease, %	4.2	4.0	4.3	4.2	4.1	2.3	<0.443	4.8	3.7	3.3	3.0	3.0	2.6	0.011
Cancer, %	0.9	1.0	1.1	0.8	0.5	0.4	0.407	1.8	1.9	1.3	1.9	1.7	1.8	0.157
Hypertension, %	22.2	20.1	20.1	19.9	16.8	17.7	0.002	24.3	24.2	22.4	20.1	20.0	18.5	<0.001
Diabetes, %	6.6	6.5	6.9	6.6	6.0	6.0	0.835	6.3	4.5	3.6	3.4	3.2	3.2	<0.001
Kidney disease, %	4.5	4.6	4.1	4.1	3.9	2.4	0.028	4.6	5.1	5.2	5.3	5.2	4.8	0.907

Table 2 shows sex-specific age-adjusted multivariable HRs (95% CIs) for all-cause mortality according to each modifiable healthy lifestyle component. All modifiable healthy lifestyles were associated with a decreased risk of all-cause mortality, except for fish and fruit intake for men and milk intake for women. The number of modifiable healthy lifestyles were inversely associated with the risk of all-cause mortality among both men and women (*P* for trend < 0.001; Supplementary Table 1, supplementary data are available in *Age and Ageing* online). Table 2 shows lifetime gain for each modifiable healthy lifestyle component at age 40 years. The lifetime gains (95% CIs) were ~2 and 5 years for ethanol intake <46.0 g/day in men and women, respectively; 4 years for never having smoked (both men and women) and 1.3–1.7 years for BMI 21–25 kg/m^2^ and a sleep duration of 5.5–7.4 h/day in both men women. The remaining modifiable lifestyles showed small but significant lifetime gains of 0.5–1.1 years.

**Table 2:** Sex-specific age-adjusted and multivariable HRs and 95%CIs of all-cause mortality for each of health lifestyles and lifetime gains (95% CI) at the age of 40 years

Men					
	Pearson-years	No. of deaths/ No. at risk	Age-adjusted HR (95% CI)	Multivariable HR (95%CI)	Lifetime gain (95% CI) at age 40 years
Subjects					
Fruits ≥1/day	140,005	2,329/8,741	0.93 (0.88–0.98)	0.97 (0.91–1.02)	0.5 (0.3–0.7)
Fish ≥1/day	84,596	1,504/5,201	1.02 (0.96–1.08)	1.02 (0.96–1.08)	0.6 (0.3–0.9)
Milk almost every day	134,576	2,257/8,407	0.91 (0.86–0.96)	0.94 (0.89–0.99)	1.1 (0.8–1.5)
Habitual exercise and/or walking	169,719	2,845/10,414	0.95 (0.90–1.00)	0.94 (0.89–0.99)	0.4 (0.0–0.8)
Body-mass index 21–25 kg/m^2^	178,526	2,581/10,947	0.84 (0.80–0.88)	0.84 (0.80–0.89)	1.3 (1.0–1.6)
Ethanol intake <46.0 g/day	222,165	3,711/13,910	0.83 (0.78–0.88)	0.87 (0.82–0.93)	1.9 (1.5–2.4)
Never having smoked,	71,159	857/4,264	0.66 (0.61–0.71)	0.68 (0.63–0.73)	3.8 (3.4–4.2)
Sleep duration 5.5–7.4 h/day	165,522	2,138/10,168	0.85 (0.80–0.90)	0.87 (0.82–0.92)	1.4 (1.0–1.8)
Women					
Subjects	Pearson-years	No. of deaths/ No. at risk	Age-adjusted HR (95% CI)	Multivariable HR (95%CI)	Lifetime gain (95% CI) at age 40 years
Fruits ≥1/day	268,534	2,120/16,123	0.84 (0.79–0.90)	0.87 (0.81–0.93)	0.5 (0.3–0.7)
Fish ≥1/day	119,971	928/6,921	0.86 (0.80–0.92)	0.89 (0.83–0.96)	1.1 (0.8–1.4)
Milk almost every day	208,767	1,734/12,525	0.94 (0.88–1.00)	0.98 (0.92–1.05)	0.5 (0.3–0.7)
Habitual exercise and/or walking	230,741	1,927/13,597	0.92 (0.86–0.98)	0.92 (0.86–0.98)	0.6 (0.3–0.9)
Body-mass index 21–25 kg/m^2^	224,552	1,630/13,336	0.83 (0.77–0.88)	0.84 (0.79–0.90)	1.7 (1.2–2.2)
Ethanol intake <46.0 g/day	434,029	3,653/26,040	0.57 (0.40–0.81)	0.65 (0.45–0.93)	4.9 (4.4–5.3)
Never having smoked	413,034	3,390/24,706	0.64 (0.57–0.72)	0.67 (0.59–0.75)	3.7 (3.2–4.2)
Sleep duration 5.5–7.4 h/day	281,623	1,739/16,709	0.81 (0.76–0.86)	0.82 (0.76–0.87)	1.6 (1.2–2.0)

Multivariable HR and Lifetime gain were adjusted further for other induvial modifiable healthy lifestyles and educational level, family history of cardiovascular disease.

**Figure 1:**
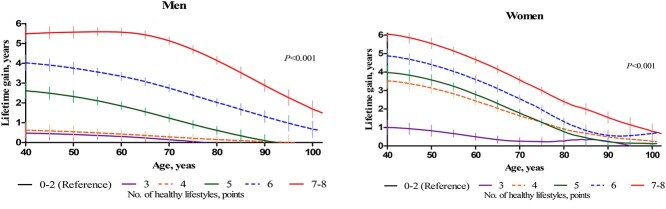
The estimation for lifetime gain and 95% CI between the age of 40 and 102 years according to the number of modifiable healthy lifestyles. Impact estimations were adjusted by educational level and family history of cardiovascular disease.

**Figure 2:**
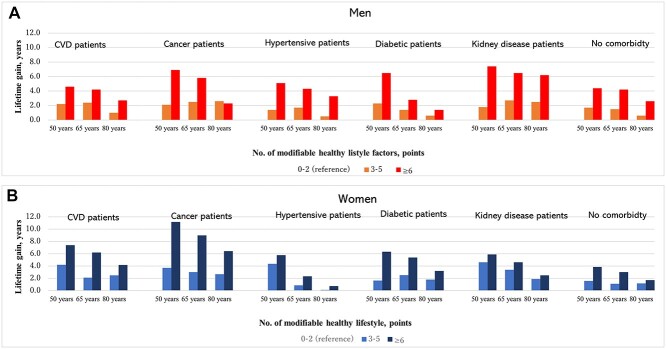
The estimation of lifetime gains at the age of 50, 65 and 80 years according to modifiable healthy lifestyles among patients with major comorbidities and without them. Lifetime gains were adjusted by educational level and family history of cardiovascular disease. Number of healthy lifestyles (0–2 points) was used as a reference.

**Figure 3:**
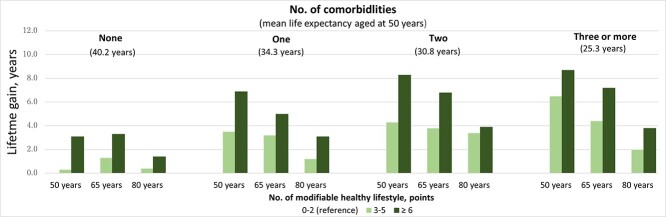
The estimation of lifetime gains at the age of 50, 65 and 80 years according to the number of modifiable healthy lifestyles among patients with none, single, double and triple or more comorbidities. Lifetime gains were adjusted by sex, educational level and family history of cardiovascular disease. Number of healthy lifestyles (0–2 points) was used as a reference. Mean age (standard deviation) at baseline were as follows; None; 54.6 (9.6) years, one; 59.8 (9.3) years, two 62.4 (8.9) years and three or more 65.7 (7.8) years. Sex-stratified analysis was not examined due to statistical under-power.

The remaining life expectancy was positively associated with the number of modifiable healthy lifestyles in a dose–response relationship at each age point for both men and women. Notably, life expectancy at 40 years (95% CI) for 7–8 points was 46.8 (45.6–48.1; *P* < 0.001) and 51.3 (50.0–52.6) years for men and women, respectively (*P* < 0.001; Supplementary Table 2, supplementary data are available in *Age and Ageing* online). The lifetime gain of the healthiest was ~6 years at age 40 years. Compared with women, the benefit of healthy lifestyles was more pronounced in the older age group for men. The potential benefit of a modifiable healthy lifestyle on lifetime gain persisted at age 80 years among men scoring ≥ 5 points and women scoring ≥ 4 points (Figure 1). The impact of healthy lifestyles also persisted among patients with CVD, cancer, hypertension, diabetes and kidney disease from middle age onwards, for both men and women (Figure 2 and Supplementary Table 3, supplementary data are available in *Age and Ageing* online). The benefits of healthy lifestyle adoption were an increased number of comorbidities, although the life expectancies of individuals with comorbidities at age 50 years were shorter than that of those with no comorbidity. In detail, the life expectancy at age 50 years among patients with no comorbidity, one, two, and three or more multimorbidities was 40.2, 34.3, 30.8 and 25.3 years, respectively, and their lifetime gains at healthy lifestyles ≥6 points were 3.1, 6.9, 8.3 and 8.7 years, respectively (Figure 3).

We observed no material difference between the included and excluded population in the current analysis at age-adjusted baseline characteristics (Supplementary Table 4, supplementary data are available in *Age and Ageing* online), average life expectancies between ages 50 and 102 years (Supplementary Figure 1, supplementary data are available in *Age and Ageing* online), and lifetime gain at age 40 years in each healthy lifestyle (Supplementary Table 5, supplementary data are available in *Age and Ageing* online). The proportions of changing rates in healthy lifestyles between baseline and 5 years of follow-up were generally small (0–25%).

The comparison of lifetime survival probabilities between our cohort and national Japanese census data are shown in Supplementary Figure 2, supplementary data are available in *Age and Ageing* online. Survival curves between our total cohort population and Japanese national census were well matched across ages 40 and 95 years for both men and women.

## Discussion

In this median 21-year population-based prospective study, we examined the association between modifiable healthy lifestyle components and lifetime gain in the general Japanese population. Consequently, the impact of a modifiable healthy lifestyle on increased lifetime persisted beyond the age of 80 years or more, for both men and women with the adoption of at least six modifiable health lifestyle behaviours. These benefits were prominent, regardless of the presence of a major comorbidity and/or multimorbidity, at each life stage since middle age.

The impact of healthy lifestyle adoption on lifetime gain was more pronounced among patients with multimorbidities throughout middle to older age. Our findings extend previous studies that suggested a total lifetime difference of patients with CVD, according to lifestyle status at middle age [28, 29]. In contrast, the Nurses’ Health Study and the Health Professionals Follow-up Study revealed no significant difference in life expectancies after the diagnosis of diabetes, CVD and cancer, as per the healthy lifestyle status among American participants (73,169 women and 38,366 men; [30]). However, unlike our study, they did not examine healthy lifestyle behaviours after those diagnoses.

This study found a significant, albeit small, impact of lifestyle on lifetime increases at middle age. However, life expectancy at age 40 years for the healthiest individuals was 46.8 and 51.3 years for men and women, respectively. These results are much higher than those of previous studies. For instance, Dutch participants without any of the three unhealthy lifestyles (smoking, hypertension and overweight) at age 45 years lived 6 years longer than those with all of them (age at death: 77.4 versus 83.4 years) [20]. Adopting four unhealthy lifestyle behaviours at middle age was associated with a reduced life expectancy of 9.2 and 9.7 years (life expectancy at 50 years: 30.4 and 34.9 years for healthiest men and women, respectively) in Norwegian men and women, respectively. Moreover, the individual impacts of being overweight, heavy alcohol intake, currently smoking and physical inactivity were −1.4, −1.4, −4.2 and −3.2 years for men and −1.5, −0.9, −4.3 and −3.5 years for women, respectively [21]. Further, four unhealthy lifestyle behaviours (heavy smoking, obesity, heavy alcohol drinking and massive red meat consumption) reduced life expectancy by 14 years in middle aged British people [17]. However, although previous studies were conducted in countries with national life expectancies of <85 years, little is known regarding ageing countries with higher life expectancy, such as Japan [2, 3]. Our findings provide novel insights into the modifiable healthy lifestyle that is universally gained during a lifetime from middle age onwards, and an average life expectancy that could be achieved at around age over 85 and over 90 years for men and women, respectively.

Adopting modifiable healthy lifestyle behaviours could potentially lower all-cause mortality in old age. The European Healthy Ageing Longitudinal Study included 2,332 Europeans aged 70–90 years and found that four healthy lifestyle components (non-smoking, light or moderate alcohol consumption, physical activity and Mediterranean diet) were associated with >50% lower risk of all-cause mortality during the 12-year follow-up [31]. In the Kungsholmen Project (1,810 Swedish men and women, aged ≥75 years), the difference in the median survival of people with non-smoking, moderate alcohol consumption, appropriate body weight, leisure-time physical activity and rich social network was −0.7, 0.5, −0.1, 1.4 and 1.9 years, respectively. The median survival of individuals with all five healthy lifestyle behaviours was 4–6 years longer than those without these behaviours [23]. Moreover, even among participants aged ≥ 85 years, the median age at death was 4 years higher among people with healthy lifestyle behaviours than those without them. However, these two studies did not examine how differences in sex and two or more comorbidities affect the potential benefits of modifiable healthy lifestyles. Our study extends the evidence by demonstrating that the benefit of healthy lifestyles was more pronounced in men than women in the older age group.

In addition, the largest lifetime gain was observed for ethanol intake in women but not men. The mechanisms underlying this sex difference merit further discussion. First, the biological mechanism underlying the lower liver function in women, as opposed to men, causes poor alcohol metabolism [32], and is associated with a higher risk of mortality from myocardial infarction, stroke, cancer and all-cause in women [33]. Second, heavy alcohol intake might be a surrogate marker of a higher socioeconomic status (SES) among men, but not women, in Japan [34]. Even after adjusting for the impact of educational level, the influence of other SES indicators, such as annual household income and occupational status, on lifetime elongation might be uncontrolled in our analysis.

Survival probabilities between our cohort data overlapped, suggesting that our results and the current Japanese national census data [27] are nationally representative, even though the baseline survey was conducted in the 1990s. The indicating health benefits regarding lifetime gain or life expectancy could provide practical metrics for health professionals, the general population and health policymakers [35]. Therefore, our findings could contribute significantly to planning future healthcare settings, public health approaches and policies relevant to ageing and industrialised countries.

### Strengths and limitations

This study is crucial as it included 40,000 participants and long-term follow-up, covered a broad geographical area in Japan, and used a validated lifestyle questionnaire. As for its limitations, first, we could not confirm causality between modifiable healthy lifestyles and all-cause mortality as this was an observational study. Second, as our study participants were Japanese, it is unclear whether our results would apply to other countries and cultures. Third, we excluded 61,564 (55.7%) participants due to a lack of modifiable healthy lifestyle information. Our findings were thus vulnerable to sampling bias. However, we found similar baseline characteristics between the excluded and included populations. Finally, we assessed healthy lifestyles at baseline survey and limited lifestyles at 5-years of follow-up, which is likely to have caused the misclassification. However, changes in limited healthy lifestyles during the 5-year period were not large.

## Conclusion

The impact of a modifiable healthy lifestyle showed a clear dose–response relationship, with a longer remaining life expectancy and lifetime gain, which persisted even age of 80 years or more among men and women. These benefits were more prevalent among patients with major comorbidities, such as CVD, cancer, hypertension, diabetes, and/or kidney disease and those with multimorbidity in each life stage since middle age. Thus, our findings underline the importance of modifiable healthy lifestyle improvements for lifespan increases in global ageing.

## Supplementary Material

aa-21-1514-File002_afac080
