# Supplementary material for: Impact of modifiable healthy lifestyle adoption on lifetime gain from middle to older age
Source: Age Ageing. 2022 May 11;51(5):afac080. doi: 10.1093/ageing/afac080 (PMC9092121; doi:10.1093/ageing/afac080)
Supplement: aa-21-1514-File002_afac080 [file aa-21-1514-file002_afac080.docx]

Impact of modifiable healthy lifestyle adoption on lifetime gain from middle to older age

**SUPPLEMENTARY MATERIAL**

- **Supplementary Table 1.** The sex specific hazard ratios (HRs) and 95% confidence intervals (95%CIs) for all-cause mortality according to the number of modifiable healthy lifestyles.
- **Supplementary Table 2.** Sex-specific remaining life expectancy according to modifiable healthy lifestyles at the age of 40, 50, 60, 80, and 85 years.
- **Supplementary Table 3.** The estimation of lifetime gains at the age of 50, 65, and 80 years according to modifiable healthy lifestyles among patients with major comorbidities and without them.
- **Supplementary Table 4.** The comparison of age-adjusted baseline characteristics and mortality rates between the included and excluded populations.
- **Supplementary Table 5.** Sex-specific age-adjusted and multivariable hazard ratios (HRs) and 95% confidence intervals (CIs) of all-cause mortality according to each of healthy lifestyles and lifetime gain (95% CI) at the age of 40, among the excluded (n=24,942 for men and 36,622 for women).
- **Supplementary Figure 1.** The comparison of average life expectancy between the ages of 50 and 102 years among men and women, according to included (20,373 men and 26,247 women), or excluded (24,942 for men and 36,622 for women) population in the current analysis.
- **Supplementary Figure 2.** Sex-specific comparison of lifetime survival probability for total cohort population and the national census data in 2018 [27].

Supplementary Table 1. The sex specific hazard ratios (HRs) and 95% confidence intervals (95%CIs) for all-cause mortality according to the number of modifiable healthy lifestyles.

|  | No. of modifiable healthy lifestyles, points | | | | | |  |
| --- | --- | --- | --- | --- | --- | --- | --- |
|  | 0-2 | 3 | 4 | 5 | 6 | 7-8 | *P* for trend |
| Men |  |  |  |  |  |  |  |
| Pearson-years | 89,724 | 127,346 | 178,700 | 181,727 | 126,194 | 60,605 |  |
| No. of deaths | 1,380 | 1,354 | 1,362 | 782 | 316 | 89 |  |
| No. at risk | 5,763 | 7,996 | 10,997 | 10,953 | 7,428 | 3,493 |  |
| Age-adjusted HR (95%CI) | Reference | 0.88 (0.81-0.94) | 0.84 (0.78-0.91) | 0.67 (0.62-0.73) | 0.61 (0.54-0.68) | 0.50 (0.41-0.62) | <0.001 |
| Multivariable-HR (95%CI) | Reference | 0.88 (0.82-0.96) | 0.85 (0.78-0.92) | 0.68 (0.62-0.75) | 0.60 (0.53-0.69) | 0.53 (0.42-0.67) | <0.001 |
| Women |  |  |  |  |  |  |  |
| Pearson-years | 11,660 | 45,086 | 97,946 | 126,870 | 102,238 | 53,506 |  |
| No. of deaths | 182 | 587 | 922 | 998 | 674 | 320 |  |
| No. at risk | 757 | 2,845 | 5,962 | 7,603 | 6,010 | 3,070 |  |
| Age-adjusted HR (95%CI) | Reference | 1.00 (0.84-1.18) | 0.76 (0.65-0.89) | 0.68 (0.58-0.80) | 0.60 (0.51-0.71) | 0.55 (0.46-0.67) | <0.001 |
| Multivariable-HR (95%CI) | Reference | 0.97 (0.82-1.17) | 0.74 (0.63-0.89) | 0.67 (0.57-0.80) | 0.62 (0.52-0.74) | 0.56 (0.46-0.69) | <0.001 |

Multivariable-HR were adjusted for age, educational level, and family history of cardiovascular disease.

Supplementary Table 2. Sex-specific remaining life expectancy according to modifiable healthy lifestyles at the age of 40, 50, 60, 80, and 85 years.

|  | No. of modifiable healthy lifestyles, points | | | | | |
| --- | --- | --- | --- | --- | --- | --- |
|  | 0-2 | 3 | 4 | 5 | 6 | 7-8 |
| Men | Remaining life expectancy (95% CI), years | | | | | |
| Aged 40 years | 41.2 (40.0-42.4) | 42.1 (40.9-43.3) | 42.1 (40.9-43.3) | 44.4 (43.2-45.6) | 45.8 (44.6-47.0) | 46.5 (45.3-47.7) |
| 50 years | 32.4 (31.2-33.6) | 32.9 (31.7-34.1) | 33.0 (31.8-34.2) | 34.7 (33.5-35.9) | 36.3 (35.1-37.5) | 38.1 (36.9-39.3) |
| 60 years | 23.7 (22.5-24.9) | 23.9 (22.7-25.1) | 24.1 (22.9-25.3) | 25.7 (24.5-26.9) | 27.0 (25.8-28.2) | 29.2 (28.0-30.4) |
| 70 years | 15.8 (14.6-17.0) | 15.8 (14.6-17.0) | 16.2 (15-17.4) | 17.4 (16.2-18.6) | 18.6 (17.4-19.8) | 21.0 (19.8-22.2) |
| 80 years | 9.6 (8.4-10.8) | 9.6 (8.4-10.8) | 9.6 (8.4-10.8) | 10.2 (9.0-11.4) | 11.5 (10.3-12.7) | 14.0 (12.8-15.2) |
| 85 years | 7.3 (6.4-8.2) | 7.3 (6.4-8.2) | 7.2 (6.3-8.3) | 7.3 (6.4-8.2) | 8.6 (7.7-9.5) | 11.5 (10.6-12.4) |
|  | No. of modifiable healthy lifestyles, points | | | | | |
|  | 0-2 | 3 | 4 | 5 | 6 | 7-8 |
| Women | Remaining life expectancy (95% CI), years | | | | | |
| Aged 40 years | 45.1 (44.9-47.3) | 46.2 (45.0-47.4) | 48.6 (47.4-49.8) | 49.1 (47.9-50.3) | 50.0 (48.8-51.2) | 51.3 (50.1-52.5) |
| 50 years | 35.6 (35.2-37.6) | 36.6 (35.4-37.8) | 38.9 (37.7-40.1) | 39.5 (38.3-40.7) | 40.2 (39.0-41.4) | 41.3 (40.1-42.5) |
| 60 years | 27.3 (25.9-28.3) | 27.8 (26.6-29.0) | 29.6 (28.4-30.8) | 30.0 (28.8-31.2) | 30.8 (29.6-32.0) | 31.9 (30.7-33.1) |
| 70 years | 18.8 (17.1-19.5) | 19.1 (17.9-20.3) | 20.7 (19.5-21.9) | 20.9 (19.7-22.1) | 21.7 (20.5-22.9) | 22.7 (21.5-23.9) |
| 80 years | 12.1 (8.5-12.9) | 12.4 (10.2-14.6) | 12.8 (12.6-15.0) | 12.6 (12.4-14.8) | 13.3 (11.1-14.9) | 14.3 (13.1-15.5) |
| 85 years | 9.2 (8.8-9.6) | 9.5 (8.6-10.4) | 9.5 (8.6-10.4) | 9.2 (8.3-10.4) | 9.7 (8.8-10.6) | 10.6 (9.7-11.6) |

Supplementary Table 3. The estimation of lifetime gains at the age of 50, 65, and 80 years according to modifiable healthy lifestyles among patients with major comorbidities and without them.

|  | Men | | | Women | | |
| --- | --- | --- | --- | --- | --- | --- |
|  | No. of healthy lifestyles, points | | | No. of healthy lifestyles, points | | |
|  | 0-2 | 3-5 | ≥6 | 0-2 | 3-5 | ≥6 |
| Cardiovascular disease patients | Lifetime gain (95% CI), years | | | Lifetime gain (95% CI), years | | |
| Age at 50 years | Reference | 1.7 (1.0-2.4) | 4.6 (3.9-5.3) | Reference | 6.1 (5.4-6.8) | 9.7 (9.0-10.4) |
| Age at 65 years | Reference | 2.4 (1.2-3.6) | 4.2 (3.1-5.3) | Reference | 4.5 (3.3-5.7) | 7.6 (6.5-8.7) |
| Age at 80 years | Reference | 1.0 (-0.4-2.5) | 2.7 (0.9-4.5) | Reference | 2.5 (1.1-4.0) | 4.2 (2.4-6.0) |
| Cancer patients | Lifetime gain (95% CI), years | | | Lifetime gain (95% CI), years | | |
| Age at 50 years | Reference | 2.1 (0.4-3.8) | 6.9 (5.9-7.9) | Reference | 3.1 (1.4-4.8) | 11.2 (10.2-12.2) |
| Age at 65 years | Reference | 2.5 (0.5-4.5) | 5.8 (4.5-7.2) | Reference | 3.0 (1.0-5.0) | 9.0 (7.7-10.4) |
| Age at 80 years | Reference | 2.6 (0.2-5.1) | 2.3 (0.3-2.4) | Reference | 2.7 (0.3-5.2) | 6.4 (4.4-6.5) |
| Hypertensive patients | Lifetime gain (95% CI), years | | | Lifetime gain (95% CI), years | | |
| Age at 50 years | Reference | 1.4 (0.9-2.0) | 5.1 (4.4-5.8) | Reference | 4.4 (3.9-5.0) | 5.8 (5.1-6.5) |
| Age at 65 years | Reference | 1.7 (0.8-2.8) | 4.3 (3.0-5.7) | Reference | 0.9 (0.0-2.0) | 2.3 (1.0-3.7) |
| Age at 80 years | Reference | 0.5 (-1.9-3.0) | 3.3 (1.8-4.7) | Reference | 0.1 (-2.3-2.6) | 0.8 (-0.7-2.2) |
| Diabetic patients | Lifetime gain (95% CI), years | | | Lifetime gain (95% CI), years | | |
| Age at 50 years | Reference | 2.3 (1.6-3.0) | 6.5 (5.2-7.8) | Reference | 1.6 (0.9-2.3) | 6.3 (4.8-7.8) |
| Age at 65 years | Reference | 1.4 (0.3-2.5) | 2.8 (1.2-4.5) | Reference | 2.5 (1.4-3.6) | 5.4 (3.5-7.3) |
| Age at 80 years | Reference | 0.6 (-1.2-2.4) | 1.4 (0.0-2.8) | Reference | 1.8 (0.0-3.6) | 3.2 (1.1-5.3) |
| No comorbidities citizen | Lifetime gain (95% CI), years | | | Lifetime gain (95% CI), years | | |
| Age at 50 years | Reference | 1.7 (1.4-2.0) | 4.4 (4.1-4.7) | Reference | 1.6 (1.3-1.9) | 3.9 (3.6-4.2) |
| Age at 65 years | Reference | 1.5 (1.0-4.0) | 4.2 (3.7-6.7) | Reference | 1.1 (0.6-3.6) | 3.0 (2.5-5.5) |
| Age at 80 years | Reference | 0.7 (0.1-1.4) | 2.7 (1.6-3.6) | Reference | 1.2 (0.6-1.9) | 1.7 (0.6-2.7) |

Lifetime gains were adjusted by educational level and family history of cardiovascular disease. No. of healthy lifestyles (0-2 points) was used as a reference.

Supplementary Table 4. The comparison of age-adjusted baseline characteristics and mortality rates between the included and excluded populations.

|  | Men | | Women | |
| --- | --- | --- | --- | --- |
|  | Included | Excluded | Included | Excluded |
| **Subjects** |  |  |  |  |
| No. at risk, n | 21,453 | 24,942 | 27,568 | 36,622 |
| Mortality rates during follow-up, % | 30.2 | 35.7 | 17.1 | 19.8 |
| Age, years (standard deviation) | 56.1 (10.0) | 58.9 (10.3) | 56.3 (9.8) | 59.1 (10.1) |
| Family history of cardiovascular disease, % | 42.4 | 44.5 | 42.7 | 45.5 |
| High perceived mental stress, % | 23.8 | 21.5 | 20.1 | 20.0 |
| College or higher education, % | 19.2 | 14.7 | 10.8 | 9.0 |
| **Comorbidities** |  |  |  |  |
| Hypertension, % | 20.4 | 18.7 | 21.7 | 20.8 |
| Diabetes, % | 6.5 | 6.0 | 3.7 | 3.8 |
| Cancer, % | 1.0 | 0.8 | 1.8 | 1.5 |
| Cardiovascular disease, % | 4.3 | 5.5 | 3.2 | 4.0 |
| Kidney disease, % | 4.3 | 3.6 | 5.2 | 4.4 |
| **No. of averrable healthy lifestyle information and its proportion** | |  |  |  |
| Fruits intake, n (%) | 21,453 (100.0) | 8,441 (33.8) | 27,568 (100.0) | 13,918 (38.0) |
| Fish intake, n (%) | 21,453 (100.0) | 18,839 (75.5) | 27,568 (100.0) | 28,412 (77.6) |
| Milk intake, n (%) | 21,453 (100.0) | 20,399 (81.8) | 27,568 (100.0) | 30,670 (83.7) |
| Habitual exercise and/or walking, n (%) | 21,453 (100.0) | 12,579 (50.4) | 27,568 (100.0) | 18,375 (50.2) |
| Body mass index, n (%) | 21,453 (100.0) | 22,484 (90.1) | 27,568 (100.0) | 32,467 (88.7) |
| Ethanol intake, n (%) | 21,453 (100.0) | 15,375 (61.6) | 27,568 (100.0) | 23,878 (65.2) |
| Smoking status, n (%) | 21,453 (100.0) | 22,360 (89.6) | 27,568 (100.0) | 27,794 (75.9) |
| Sleep duration, n (%) | 21,453 (100.0) | 22,495 (90.2) | 27,568 (100.0) | 32,560 (88.9) |

Supplementary Table 5. Sex-specific age-adjusted and multivariable hazard ratios (HRs) and 95% confidence intervals (CIs) of all-cause mortality according to each of healthy lifestyles and lifetime gain (95% CI) at the age of 40, among the excluded (n=24,942 for men and 36,622 for women).

| Men |  |  |  |  |  |
| --- | --- | --- | --- | --- | --- |
| Subjects vs. others (Reference) | Pearson-years | No. of deaths/ No. at risk | Age-adjusted  HR (95% CI) | Multivariable  HR (95%CI) | Lifetime gains (95% CI)  age at 40 years |
| Fruits >1/day | 55,941 | 1,302/3,568 | 0.87 (0.81-0.93) | 0.89 (0.83-0.96) | 1.3 (1.1-1.5) |
| Fish >1/day | 78,694 | 2,017/4,986 | 0.99 (0.94-1.04) | 1.00 (0.95-1.05) | 0.2 (0.0-0.4) |
| Milk almost every day | 126,518 | 3,346/8,156 | 0.96 (0.92-1.01) | 0.99 (0.95-1.04) | 0.5 (0.2-0.8) |
| Habitual exercise and/or walking | 102,496 | 2,270/6,450 | 0.79 (0.74-0.83) | 0.78 (0.74-0.83) | 1.9 (1.4-2.3) |
| Body mass index 21-25 kg/m^2^ | 187,424 | 3,957/11,639 | 0.86 (0.83-0.90) | 0.87 (0.83-0.91) | 1.7 (1.3-2.1) |
| Ethanol intake <46.0 g/day | 168,112 | 4,650/11,301 | 0.94 (0.88-1.00) | 0.96 (0.90-1.02) | 0.7 (0.2-1.2) |
| Never having smoked | 75,175 | 1,388/4,587 | 0.71 (0.67-0.75) | 0.71 (0.67-0.75) | 3.2 (2.8-3.6) |
| Sleep duration 5.5-7.4 hrs/day | 162,940 | 3,230/9,948 | 0.87 (0.83-0.91) | 0.88 (0.84-0.91) | 1.3 (0.8-1.7) |
| Women |  |  |  |  |  |
| Subjects vs. others (Reference) | Pearson-years | No. of deaths/ No. at risk | Age-adjusted  HR (95% CI) | Multivariable  HR (95%CI) | Lifetime gains (95% CI)  age at 40 years |
| Fruits >1/day | 132,093 | 1,503/8,315 | 0.85 (0.79-0.92) | 0.87 (0.81-0.94) | 1.0 (0.7-1.3) |
| Fish >1/day | 125,689 | 1,617/7,395 | 0.99 (0.94-1.05) | 1.01 (0.96-1.07) | 1.1 (0.9-1.3) |
| Milk almost every day | 223,130 | 2,950/13,715 | 0.96 (0.92-1.01) | 1.00 (0.95-1.05) | 0.3 (0.0-0.6) |
| Habitual exercise and/or walking | 154,739 | 1,750/9,529 | 0.80 (0.75-0.86) | 0.81 (0.76-0.86) | 1.5 (1.2-1.8) |
| Body mass index 21-25 kg/m^2^ | 268,743 | 2,772/15,998 | 0.83 (0.79-0.88) | 0.84 (0.80-0.89) | 1.4 (1.0-1.8) |
| Ethanol intake <46.0 g/day | 385,488 | 5,248/23,762 | 0.87 (0.54-1.40) | 1.01 (0.63-1.62) | 4.1 (3.6-4.6) |
| Never having smoked | 428,600 | 5,247/25,549 | 0.63 (0.58-0.68) | 0.64 (0.59-0.70) | 3.2 (2.7-3.6) |
| Sleep duration 5.5-7.4 hrs/day | 312,055 | 3,076/18,249 | 0.84 (0.80-0.88) | 0.85 (0.81-0.89) | 1.3 (0.9-1.7) |

Multivariable HRs were adjusted for age, educational level, and family history of cardiovascular disease.

Supplementary Figure 1. The comparison of average life expectancy between the ages of 50 and 102 years among men and women, according to included (20,373 men and 26,247 women), or excluded (24,942 for men and 36,622 for women) population in the current analysis.

Supplementary Figure 2. Sex-specific comparison of lifetime survival probability for total cohort population and the national census data in 2018 [27].
